# Ethanolamine Utilization in Bacteria

**DOI:** 10.1128/mBio.00066-18

**Published:** 2018-02-20

**Authors:** Karan Gautam Kaval, Danielle A. Garsin

**Affiliations:** a Department of Microbiology and Molecular Genetics and The UT Center for Antimicrobial Resistance and Microbial Genomics, McGovern Medical School, The University of Texas Health Science Center at Houston, Texas, USA; UT Southwestern Med Center Dallas

**Keywords:** catabolism, ethanolamine, gene regulation, microbial pathogenesis

## Abstract

Ethanolamine (EA) is a valuable source of carbon and/or nitrogen for bacteria capable of its catabolism. Because it is derived from the membrane phospholipid phosphatidylethanolamine, it is particularly prevalent in the gastrointestinal tract, which is membrane rich due to turnover of the intestinal epithelium and the resident microbiota. Intriguingly, many gut pathogens carry the *eut* (ethanolamine utilization) genes. EA utilization has been studied for about 50 years, with most of the early work occurring in just a couple of species of *Enterobacteriaceae*. Once the metabolic pathways and enzymes were characterized by biochemical approaches, genetic screens were used to map the various activities to the *eut* genes. With the rise of genomics, the diversity of bacteria containing the *eut* genes and surprising differences in *eut* gene content were recognized. Some species contain nearly 20 genes and encode many accessory proteins, while others contain only the core catabolic enzyme. Moreover, the *eut* genes are regulated by very different mechanisms, depending on the organism and the *eut* regulator encoded. In the last several years, exciting progress has been made in elucidating the complex regulatory mechanisms that govern *eut* gene expression. Furthermore, a new appreciation for how EA contributes to infection and colonization in the host is emerging. In addition to providing an overview of EA-related biology, this minireview will give special attention to these recent advances.

## INTRODUCTION

All bacterial and eukaryotic cells contain the membrane lipid phosphatidylethanolamine, which can be a source of the metabolically useful compound ethanolamine (EA) (1, 2). EA arises when phosphodiesterases break down phosphatidylethanolamine into glycerol and EA (3, 4). The animal gut provides a rich, natural source of EA due to the host diet and the residential microbiota (1, 2, 5). Not surprisingly, bacteria capable of catabolizing EA, which can serve as a valuable source of carbon and nitrogen, are found in the mammalian gut and include species of *Enterococcus*, *Escherichia*, *Clostridium*, *Listeria*, *Klebsiella*, and *Salmonella*. However, the capability to utilize EA is not limited to this niche and is found in species of *Erwinia*, *Flavobacterium*, *Mycobacterium*, *Fusobacterium*, and *Corynebacterium*, to name a few (6, 7).

The ability to catabolize EA is encoded by the ethanolamine utilization (*eut*) genes, the most central being *eutB* and *eutC*, which together comprise the two subunits of the ethanolamine ammonia lyase. EutBC breaks EA down into the gases acetaldehyde and ammonia, which serve as sources of carbon and nitrogen, respectively (5, 8–11). In addition to the ammonia lyase, a variety of accessory genes that enhance EA breakdown can be found, usually together in an operon. These operons vary greatly in complexity. In some species, they are short and include just a few genes, whereas in others they are long: the *Enterococcus faecalis* locus contains 19 genes, for example (7, 12, 13).

The goal of this review is to highlight the significant recent advances made in understanding EA utilization. While the functions encoded by many of the *eut* genes were elucidated years ago, there was recent progress in grasping some of the more recalcitrant. Additionally, tremendous headway was achieved in understanding how the *eut* genes are regulated. Since generation of close to 20 proteins dedicated to a single process is an energy-intensive commitment, it is perhaps not surprising that the *eut* genes are tightly regulated in a complex manner. Finally, while strong connections between EA utilization and host-pathogen interactions have been noted for years, detailed and mechanistic comprehension is only now emerging thanks to recent studies.

## ETHANOLAMINE CATABOLISM

### Catabolic reactions.

**FIG 1:**
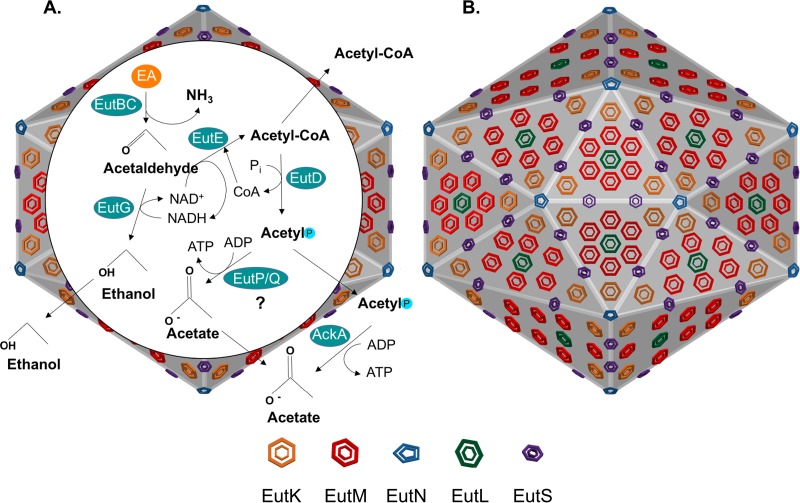
Eut protein functions. (A) Eut enzymatic proteins that catabolize EA. (B) Eut structural proteins that comprise the BMC. See the text for details.

The first studies of EA utilization by bacteria were performed using *Escherichia coli* or *Salmonella enterica* serovar Typhimurium and involved biochemical purification and study of a newly discovered AdoCbl-dependent ethanolamine ammonia lyase (10, 14, 15). Later, a transposon mutagenesis screen of *S*. Typhimurium mutants unable to utilize EA identified the genes that encode this enzyme and some of the others involved in EA catabolism. (See Fig. 1A for an overview of the *eut* genes’ encoded functions and in which enzymatic reactions they participate.) The two subunits that comprise the ammonia lyase activity were mapped to the *eutB* and *eutC* genes. The acetaldehyde dehydrogenase, which can generate acetyl coenzyme A (acetyl-CoA) from the acetaldehyde produced by EutBC, was encoded by *eutE* (5, 11). Acetyl-CoA can subsequently be used in a variety of metabolic reactions, including the tricarboxylic acid (TCA) cycle, the glyoxalate cycle, or lipid biosynthesis. Alternatively, acetyl-CoA can be a source of ATP and acetate when transformed into acetyl phosphate by a phosphotransacetylase encoded by EutD and then acetate by the housekeeping acetate kinase (AckA) (16, 17). Rather than being processed by EutE, acetaldehyde can also be converted into alcohol by EutG, an alcohol dehydrogenase (18).

As mentioned, the EutBC enzyme requires the cofactor AdoCbl for its activity. *eut* operons therefore frequently encode proteins related to meeting this requirement. For example, *eutT* encodes a corrinoid cobalamine adenosyltransferase that can transfer the adenosine from ATP to precursor corrinoids to generate AdoCbl (19, 20). The active ammonia lyase holoenzyme consists of EutB, EutC, and the cofactor AdoCbl. It is not a true enzyme in that each round of catalysis inactivates the complex by irreversible cleavage of the Co-C bond of AdoCbl. The modified cofactor must be removed or readenosylated in order for further reaction cycles to take place. EutA is a reactivating factor that removes the damaged cofactor, thereby allowing a new molecule of AdoCbl to bind (21). While *eutT* and *eutA* are usually found in the *eut* operons, bacteria that metabolize EA encode the generation (using the *cob* and *cbi* genes) or importation (using the *btu* genes) of corrinoid precursors in separate operons (22, 23). Of course, EA must be able to cross the cytoplasmic membrane to be metabolized, which it can do by diffusion at neutral pH. At acidic pH, the proportion of EA that is protonated increases and the transporters Eat or EutH are utilized (7, 24).

The functions of the proteins encoded by *eutJ*, *eutP*, and *eutQ* have long been unclear. EutJ is required for utilization of EA as a carbon, but not nitrogen, source and shares some homology to DnaK and Hsp70 chaperones. It was postulated to chaperone EutE and EutG (18, 24). *S*. Typhimurium *eutP* and *eutQ* deletion mutants grew as well as the wild type on EA, although one study noted that more acetaldehyde was released (25). However, a recent breakthrough investigation demonstrated that *eutP* and *eutQ* encode acetate kinases capable of metabolizing acetyl phosphate like AckA, described above (26). EutP and EutQ bear no resemblance to AckA at the amino acid level, though there may be conservation at the structural level of key amino acids required for substrate binding. Why do some species carry these additional acetate kinases if AckA can perform the required function? The authors show that *eutQ* is required for EA utilization under certain conditions, specifically anoxic conditions in which tetrathionate acts as the terminal electron acceptor. Additionally, EutP and EutQ might enhance EA utilization by being localized to the bacterial microcompartments (BMCs) (speculatively included in Fig. 1A) where EA catabolism takes place in some species (described below) (26).

### Microcompartment formation.

Another class of gene products that the long *eut* operons encode are structural proteins that self-assemble into BMCs and include EutS, EutM, EutK, EutL, and EutN. These proteins form a selectively permeable, icosahedral protein shell with the core EA catabolic enzymes encased inside (Fig. 1B) (27, 28). To briefly describe the *eut* BMC proteins, all oligomerize into hexamers or pseudohexamers, except for EutN (29). EutM is thought to be the most prevalent, acting as a basic building block. EutK has a mysterious C-terminal domain that forms a helix-turn-helix suggestive of nucleotide binding, though no functional role for this domain has yet been identified (30). The EutS hexamer has a bent shape that allows it to occupy the edges of the icosahedral BMC structure, while EutN oligomerizes into a pentamer that forms the vertices (29, 30). The two-domain protein, EutL, oligomerizes as a trimer, forming pseudohexamers with a very large central pore that is postulated to be gated and to allow the entry of AdoCbl and other cofactors (30–32). For more information on the fascinating structural and functional aspects of BMC proteins, see these excellent, recent reviews by Bobik et al. and Kerfeld et al. (27, 28).

Not all *eut* loci encode the structural components required for BMC formation, but for those that do, the advantages they bring to EA catabolism are thought to be 3-fold. (i) The toxic intermediate acetaldehyde is kept away from the cytoplasm. (ii) Acetaldehyde, being volatile in nature, is retained rather than lost. (iii) Acetaldehyde and other intermediates are concentrated, increasing the efficiency of the downstream reactions (25, 33). How do catabolic enzymes become encapsulated within the BMCs? Recent work suggests that they form from the inside-out with key enzymes containing short encapsulation peptides (EPs) on either their N or C termini that direct their localization (34–36). A fusion of the EP of EutC to green fluorescent protein (GFP) definitively demonstrated its role in encapsulation (37). EutE is predicted to have an EP, as well as EutD (34).

One inherent functional challenge of having the enzymes for EA utilization located inside this organelle-like protein structure is controlling the entry and exit of the substrates, cofactors, and products of the reactions. As mentioned, the BMCs appear effective at retaining acetaldehyde, and AdoCbl may be allowed to enter through a gated pore in EutL. What about the cofactors CoA and NAD+, both required by the acetaldehyde dehydrogenase EutE? It has been postulated that these cofactors are regenerated within the BMC, forming “private cofactor pools.” The alcohol dehydrogenase EutG regenerates NAD^+^ from NADH, and the phosphotransacetylase EutD regenerates CoA from acetyl-CoA (38). With new evidence that EutP and EutQ may be EA-specific acetate kinases, one interesting speculation is that they also reside in the BMC. The ATP generated by these enzymes could be used for removing the damaged corrinoid from EutBC, an ATP-requiring process carried out by EutA, and/or readenosylating the corrinoid, a reaction carried out by the adenosyl transferase EutT (26).

Overall, the catabolism of EA, particularly in those bacteria carrying long *eut* loci, is a very complex process requiring a multitude of genes that encode not only the enzymes required, but sophisticated, structural proteins. Generation of all the components necessary—close to 20 in some cases—represents a serious investment for a bacterium. Not surprisingly, intricate regulatory mechanisms have evolved to ensure that the EA genes are expressed only under appropriate conditions.

## REGULATION OF ETHANOLAMINE UTILIZATION

### EutR.

**FIG 2:**
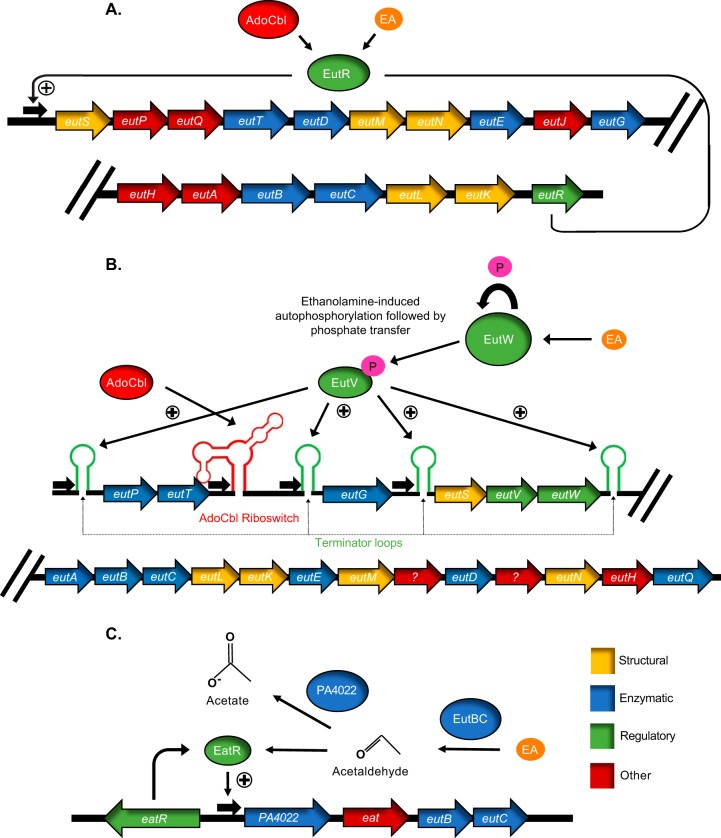
Regulation of *eut* gene expression in three characterized systems. (A) EutR (*S*. Typhimurium). (B) EutV (*E. faecalis*). (C) EatR (*P. aeruginosa*). The promoters are represented by boldface black arrows, whereas structures arising from the transcripts are shown in green (terminators) or red (riboswitch). See the text for further details.

Among the identified mutations in *S*. Typhimurium resulting in loss of EA utilization was an insertion in *eutR*, predicted to encode a positive transcriptional regulator (5, 11). Further study of EutR and the transcriptional organization of the *eut* genes in *S*. Typhimurium revealed the following facts. All of the *eut* genes are under control of one upstream promoter, P1, which is induced by activated EutR. *eutR* is the final gene carried by the locus and is preceded by P2, a low-level, constitutive promoter (5, 11). The transcriptional organization generates a positive-feedback loop in which increasing amounts of active EutR will produce more *eutR* transcripts. EutR requires the presence of both EA and AdoCbl for activity, though overexpression of EutR allows for induction by the addition of just one of these compounds (Fig. 2A) (39). Since EutR, at least at low levels, requires AdoCbl for activity, and EutBC requires AdoCbl to carry out its catabolic function, it was of interest to study the competition between EutR and EutBC for AdoCbl (40). Two nonexclusive theories for maintaining EutR activity during induction of the *eut* genes were postulated. (i) Autoregulation through the P1 promoter equalizes the levels of EutR with EutBC, enabling EutR to effectively compete for AdoCbl. (ii) Activation of EutR by EA alone happens when large amounts of EutR are present, as would occur under high levels of induction.

Further work on EutR was not undertaken until recently, and the use of both genomic and biochemical approaches enhanced the understanding previously gained by genetics. For example, while the early studies had suggested direct binding of EA to EutR, recent work in enterohemorrhagic *Escherichia coli* (EHEC) presented the first biochemical evidence. Using purified EutR, direct binding of EutR to the P1 promoter was observed by electrophoretic mobility shift assays (EMSAs). Furthermore, the presence of AdoCbl increased the binding (41). Moreover, DNase I footprints verified a comparative genomics study that had identified the binding sequence by finding conserved promoter elements, which was in a region just upstream of the −35 position (7). EutR belongs to the family of AraC/XylS-type transcriptional regulators, and this data is consistent with previous studies showing that AraC/XylS-type transcriptional regulators bind at or upstream of −35 promoter elements to induce gene transcription. Binding to the P1 promoter occurred regardless of whether EA, AdoCbl, or both were added to the reaction (41). However, *in vivo*, EHEC EutR behaved as previously described for *S*. Typhimurium EutR (39); both EA and AdoCbl were required to induce expression of the *eut* genes. The authors proposed that EutR might bind its promoter in the absence of ligands, but requires the ligands to be competent for transcriptional activation (41).

One of the most important discoveries to come out of the more recent work on EutR is the finding that it regulates genes other than those in the *eut* locus. Specifically, EA added to *in vitro* cultures was observed to activate the transcription of genes associated with virulence. In EHEC, these include genes encoding important virulence regulators (*ler*, *qseC*, and *qseE*), *stx2a* (encoding Shiga toxin), and genes encoding parts of the LEE (locus of enterocyte effacement) type III secretion system, which are positively regulated by the transcriptional activator Ler. The activation of these genes was not dependent on EA metabolism: i.e., increased transcription still occurred in a *eutB* mutant. Activation of the genes occurred at almost all concentrations of EA tested, with two peaks being apparent: one in the micromolar range and one in the millimolar range. EutR was postulated to be involved in activation in the millimolar range, as its gene was not expressed at micromolar concentrations, and no defects in EA-induced gene expression were observed in a *eutR* background at the lower concentrations. The authors postulated that a different activator, not yet identified, was responsible for EA-related gene expression at the lower concentrations (42, 43).

To further understand the role of EutR, its ability to directly bind the *ler* promoter region was examined, which consists of two promoters, P1 and P2, and EutR was found to bind just upstream of the −35 position of the P2 promoter. By increasing the production of the Ler positive transcriptional regulator, EutR was surmised to indirectly upregulate the other LEE genes (41). In later work, EA was discovered to additionally influence the expression of the fimbrial genes of EHEC. EutR was required for some of these genes’ expression, but whether the regulation was direct or through EutR’s control of Ler expression was not established (44). In conclusion, EutR directly and indirectly regulates important virulence genes in EHEC. The role of ethanolamine utilization, including the EutR regulator, in the context of host adaptation will be further discussed in the final section of this review article.

### EutV.

Like the *Enterobacteriaceae*, *Firmicutes*, including some *Enterococcus*, *Streptococcus*, *Listeria*, and *Clostridium* species, contain a long *eut* locus and largely share the same genes that encode the structural and enzymatic proteins (Fig. 2B) (7). However, when the first *Firmicutes eut* locus was studied in *E. faecalis*, the lack of a *eutR* gene encoding the regulator was noted. Instead, a two-component system (TCS) consisting of a sensor histidine kinase and a response regulator, designated EutW and EutV, respectively, was found (12, 13). Like most TCSs, when activated, EutW autophosphorylates a histidine residue, and then EutV catalyzes the transfer of the phosphate group to an aspartate in its receiver domain. EA was shown to be the activating compound by *in vitro* and *in vivo* studies (12, 13, 45).

EutV is a member of the ANTAR (AmiR and NasR transcriptional antiterminator regulators) family of response regulators (46). Rather than controlling transcription by binding DNA and modulating transcription initiation, ANTAR proteins have an RNA-binding output domain that regulates by a postinitiation mechanism. The ANTAR output domain interacts with transcriptional terminators in the untranslated regions (UTRs) preceding certain *eut* open reading frames (ORFs). Specifically, the binding prevents the terminators from forming, allowing RNA polymerase (RNAP), which would otherwise dissociate from the transcript, to generate full-length transcripts. The *eut* locus of *E. faecalis* has four of these rho-independent terminators preceding *eutP*, *eutG*, *eutS*, and *eutA*. Three of these upstream regions also have constitutive promoters (13, 45, 47). Further sequence analysis uncovered a dual-hairpin structure preceding the terminator hairpins characterized by two hexanucleotide loops. Due to the second hairpin sequence overlapping the 5′ half of the sequence that comprises the terminator structure, the formation of the second hairpin was predicted to abrogate the terminator (45). By both mutational analysis and *in vitro* biochemical studies, binding of EutV to the dual-hairpin structures was demonstrated to cause antitermination (45, 48).

Notably, the two-hairpin structure of the EutV substrate is unique among those described for other antiterminator systems, which typically consist of a single hairpin (49, 50). It was postulated that the dual-hairpin structure evolved to interact with the dimeric response regulator, as these proteins often form dimers as a result of phosphotransfer. Indeed, unphosphorylated EutV was demonstrated to be in the monomeric state, whereas dimerization was favored upon phosphotransfer. Furthermore, phosphorylated and dimerized EutV bound the dual-hairpin substrate with higher affinity relative to unphosphorylated, monomeric EutV. Overall, the phospho-induced dimerization of the EutV ANTAR response regulator followed by binding to a paired sequence in RNA is analogous to response regulators that initiate transcription by binding paired sequences in DNA (45, 48).

Other *Firmicutes* carrying long *eut* loci, including *Streptococcus*, *Clostridium*, and *Listeria* species, also contain the EutV regulatory system (7, 12, 13). Not surprisingly, dual-hairpin motifs are also found in the *eut* loci of these organisms. Additionally, this substrate is associated with the genes regulated by different ANTAR proteins, including the nitrogen-sensing regulators NasR and AmiR (45, 51). A deeper understanding of the structural features of this unique regulatory complex comprised of a dimerized response regulator interacting with a dual-hairpin RNA substrate awaits further mutant analysis and ultimately an X-ray crystal structure.

Similar to *eut* operons regulated by EutR, those regulated by the EutV system also require AdoCbl. However, unlike EutR, no influence of AdoCbl on the specific activities of EutV or EutW was observed in any *in vitro* assays (12, 13, 45). Furthermore, many of the *eut* loci containing *eutV* contain another unique structural feature, a riboswitch that binds AdoCbl, as demonstrated by in-line probing assays (13). Riboswitches are structures that form in RNA transcripts and are structurally complex relative to other features—hairpin terminators for example. Riboswitch aptamers bind metabolites, and there are currently about 20 different ligand classes, including those that bind lysine and glycine, coenzymes, including AdoCbl and *S*-adenosylmethionine (SAM), and ions, such as magnesium and fluoride (reviewed in reference 52). Riboswitch aptamers are found in the UTRs of nascent transcripts and typically regulate gene expression in *cis* by affecting structures located downstream on the “expression platform.” The common mechanism is the stabilization/destabilization of hairpins: either terminator/antiterminator hairpins or hairpins that block/allow access to a ribosome binding site (RBS). By these mechanisms, riboswitches can regulate transcription or translation, respectively (52).

The AdoCbl-binding riboswitch in the *E. faecalis eut* locus is encoded in a region of about 300 bp between the *eutT* and *eutG* genes (Fig. 2B). Because of its location, about 200 bp upstream of the terminator hairpin preceding *eutG*, it was first postulated that it might regulate gene expression by controlling the stability of this terminator (13). However, the demonstration that EutV directly regulates the *eutG* terminator (45) and, moreover, the discovery that the riboswitch and the terminator are on separate transcripts (47) made this typical mechanism of *cis* regulation unlikely. The breakthrough in understanding occurred when a EutV binding site not associated with a terminator was identified downstream and on the same transcript of the AdoCbl-binding aptamer. Studies conducted with both *E. faecalis* and in *Listeria monocytogenes* discovered that this binding site and the riboswitch comprise a noncoding RNA, EutX/Rli55, which is produced under control of a constitutive promoter (53–57). Under conditions in which EA is available, but AdoCbl is not, EutX acts as a sponge that binds and sequesters active EutV. However, as revealed by Northern blotting and transcriptome sequencing (RNA-seq), the presence of AdoCbl generated a shorter form of EutX lacking the EutV binding site. The 3′ end of this shorter form of EutX was mapped to the end of the structured riboswitch aptamer. The final hairpin has structural features consistent with it being a transcriptional terminator, and *in vitro* transcription runoff assays revealed that this terminator is stabilized by AdoCbl binding (54). Hence, AdoCbl activates transcription by negatively controlling the formation of the dual-hairpin substrate in the EutX small RNA (sRNA) that normally sequesters active EutV (53–57).

Thus, in a manner completely different from the EutR regulatory system, AdoCbl also induces the expression of the *eut* genes in those bacteria containing EutV. Because the EutX sRNA acts in *trans*, it is not required to have the same transcriptional orientation as the *eut* genes and indeed is found in the reverse orientation in the *Streptococcus sanguinis eut* locus (56). In theory, EutX could exist anywhere in the genome. As mentioned, *Clostridium* species have *eut* loci that contain EutV, but do not have an obvious, associated riboswitch (13). It would be interesting to determine if gene expression in *Clostridium* also requires AdoCbl and if the mechanism involves a EutX-like sRNA located outside the *eut* locus.

### EatR.

*Pseudomonas aeruginosa* strain PAO1 contains a *eut* operon of the short variety that lacks the BMC structural components but will support the growth of *P. aeruginosa* on EA when provided as the sole source of carbon or nitrogen (58). The operon consists of four genes encoding an acetaldehyde dehydrogenase (PA4022), an EA transporter (Eat), and the two subunits, EutB and EutC, that comprise the ethanolamine ammonia lyase (Fig. 2C). Note that PA4022 is not a EutE homologue, but rather is 98% similar to ExaC, which generates acetate using NAD^+^ as a cofactor. How these *eut* genes are induced by EA was unknown, as the genome encodes neither the EutR nor the EutV regulatory system. In recent work, it was observed that the promoter upstream of the operon has a conserved −24/−12 promoter recognized by the alternative sigma factor σ^54^. The RNA polymerase (RNAP) holoenzyme formed with σ^54^, RpoN, requires an enhancer-binding protein (EBP) to isomerize from a closed to an open configuration. The investigators found such an EBP encoded immediately upstream of the *eut* operon with an open reading frame in the opposite direction. Loss of this gene (*eatR*) prevented *eut* gene induction and utilization of EA. Very interestingly, and unlike EutR and EutV, EatR is not activated by EA. Rather, it senses acetaldehyde. Several lines of evidence supported this conclusion. First, the *eut* genes could be induced with addition of acetaldehyde. They could not be induced by EA in a *eutB* background, presumably because the cells are no longer capable of producing acetaldehyde from EA. In contrast, higher levels of induction were observed in a strain lacking the acetaldehyde dehydrogenase, a background in which acetaldehyde levels are increased. Finally, acetaldehyde could induce the *P. aeruginosa eut* genes when heterologously expressed in *E. coli*. The authors postulate that sensing acetaldehyde rather than EA in a system incapable of building BMCs might have the advantage of preventing acetaldehyde toxicity: as acetaldehyde levels rise, the expression of the dehydrogenase that breaks it down also rises to counteract deleterious effects (58).

However, there are examples of short *eut* operons in *eutR*-containing bacteria, including other species of *Pseudomonas* (7, 58). Presumably, these EutR regulators also respond to EA, rather than acetaldehyde, like those studied in *E. coli* and *Salmonella*. Therefore, the need for an acetaldehyde-responsive regulator, rather than an EA-responsive regulator, does not appear to be universal in short *eut* loci. It would be interesting to switch out the EatR for the EutR regulatory system and study how this affects the kinetics and efficiency of EA utilization in *P. aeruginosa*. Perhaps the EatR system is less prone to toxicity at higher concentrations of EA than the EutR system by virtue of responding to acetaldehyde rather than EA. Another issue not addressed in the initial study of the EatR system is the role of AdoCbl (58). AdoCbl was added under all medium conditions, and whether or not it also is necessary for inducing EatR was not examined.

## ETHANOLAMINE UTILIZATION AND HOST INTERACTIONS

Many of the species that contain the *eut* genes, *Escherichia*, *Salmonella*, *Clostridium*, *Listeria*, and *Enterococcus*, reside in the gut, and with the exception of *Enterococcus faecalis* and commensal strains of *Escherichia coli* are pathogens. Overall, there is a global association of gastrointestinal (GI) pathogens with EA utilization, as discovered by a computational analysis that used literature and genome mining to predict factors connected to bacterial food poisoning (7). As mentioned, the gastrointestinal tract provides a rich source of ethanolamine; EA concentrations over 2 mM were measured from bovine intestinal content (59). In general, EA utilization has long been thought to be a likely virulence determinant, but its role has remained murky (6). A major question is whether EA simply serves as a valuable source of nitrogen and/or carbon in the host or plays a more intimate role in promoting infection. In this section, we highlight the studies that have examined the role of EA utilization as it pertains to host interactions. The focus will be on the gut pathogens *L. monocytogenes*, EHEC, and *S*. Typhimurium, about which the most is known and from which mechanistic understanding is starting to emerge.

Expression of the *eut* genes is activated in *L. monocytogenes* in a variety of host environments. Using tiling microarrays to study *L. monocytogenes* gene expression under several different conditions, one study discovered strong upregulation of the *eut* genes in the intestines of infected mice and weaker, but still significantly higher, levels of expression in human blood relative to bacteria cultured in rich medium (60). *L. monocytogenes* is an intracellular pathogen that escapes the phagosome and replicates in the cytoplasm of infected cells, including cells of the intestinal epithelium and the innate immune cells. In the host environment of human colon epithelial (Caco-2) cells, a microarray analysis found that the *eut* operon of *L. monocytogenes* was upregulated (61). Does the upregulation of the *eut* genes promote *L. monocytogenes* survival and pathogenesis in the host? While this has yet to be studied extensively, it was discovered that a *eutB* deletion mutant was defective in intracellular replication, reaching CFU a log lower than that of the isogenic wild-type strain (61). Furthermore, an intravenous (i.v.) mouse infection model documented less CFU in the spleen and liver of mice infected with a *eutB* mutant and a mutant in the AdoCbl riboswitch, which is unable to activate *eut* gene expression. There was not a significant difference between the two mutants (56). Since EutB is one of the subunits that comprise the ammonia lyase, these data suggest EA catabolism promotes survival in both intestinal epithelial cells and during bloodstream infection (56, 61).

Both EHEC and enteropathogenic *E. coli* (EPEC) are diarrhea-inducing, gastrointestinal pathogens that tightly adhere to the gut epithelium, causing a characteristic structure called an attaching and effacing lesion. The first evidence of a role for EA in this process was discovered, not for EA, but for the precursor membrane lipid, phosphatidyl-EA. EHEC and EPEC were observed to preferentially bind this membrane lipid over phosphatidylcholine (PC) and phosphatidylserine (62). Furthermore, interaction of the host cells with EHEC or EPEC induced apoptosis, which was correlated with an increase in the levels of phosphatidyl-EA in the outer leaflet (63). The increase was caused by higher rates of phosphatidyl-EA synthesis with a concomitant reduction in PC synthesis (64).

In addition to being a precursor for a membrane lipid important for EHEC host cell attachment in the intestine, Bertin et al. (59) established a role for EA as a valuable source of nitrogen in this environment. Sterilized bovine intestinal content, a biological fluid consisting of mucus, digested food, and host and microbiome debris, was utilized for their experiments. First, they measured the amount of EA naturally found in this fluid (2 mM) and then assayed for its utilization under different conditions. EHEC was able to utilize EA as a nitrogen source, and it provided a competitive advantage in this medium; the wild-type strain outcompeted *eutB* and *eutH* isogenic mutants (59). As mentioned above, the EutR regulator was shown to directly induce the expression of certain virulence genes in addition to the *eut* genes (41–44). To study the effects of EutR-regulated gene expression on the pathogenic behavior of EHEC, Kendall et al. examined pedestal formation on HeLa cells. They demonstrated that the addition of EA significantly increased pedestal formation and the number of infected cells, whereas a *eutR* mutant was significantly attenuated in comparison (43).

Studies employing a mouse typhoid model did not observe a role for the *eut* genes in *S*. Typhimurium pathogenesis (18, 65). In this model, the animals develop a typhoid fever-like disease, but acute inflammation characterized by neutrophil invasion does not develop. Inflammation in the intestine improves *S*. Typhimurium growth because it increases the availability of tetrathionate, an oxidized form of thiosulfate. Under anaerobic conditions, like those found in the gut, tetrathionate can be used as a respiratory electron acceptor in lieu of oxygen by *S*. Typhimurium, which encodes a tetrathionate reductase needed to generate this compound (66). *In vitro*, it was observed that *S*. Typhimurium requires tetrathionate in order to grow anaerobically on EA (67). Based on this knowledge, the role of EA utilization in *S*. Typhimurium pathogenesis was investigated in a mouse colitis model. In this model, mice are pretreated with streptomycin, which results in neutrophil transmigration into the intestinal lumen with concomitant inflammation. In this model, a *eutC* mutant was recovered in lower numbers and was outcompeted by the wild type when inoculated as a mixture of the two strains. The growth advantage was found to be completely dependent on the presence of *ttrA* (65). The lower growth levels resulting from loss of EA catabolism in this model were confirmed by Anderson et al., who specifically looked at a *eutB* mutant (68). Not surprisingly, loss of the *eut* locus regulator, EutR, also depressed growth in the intestine. However, it was discovered that *eutR* was more defective than *eutB* when disseminating to the spleen. These data suggested that *eutR* was contributing more to the infectious process than just EA catabolism during systemic dissemination of *S*. Typhimurium. Further experiments showed that *eutR*, but not *eutB*, *S*. Typhimurium mutants were defective in macrophage intracellular replication and systematic infection compared to the wild type. In contrast, recall that *eutB* mutants of *L. monocytogenes* were defective in these processes, suggesting that EA catabolism promotes survival of *L. monocytogenes*, but not *S*. Typhimurium, in this environment. If EA catabolism does not help *S*. Typhimurium in the intracellular environment, why is EutR protective? Anderson et al. showed that EutR regulates the genes on *Salmonella* pathogenicity island 2 (SPI-2), which is required for *S*. Typhimurium intracellular replication and survival. SPI-2 contains a type 3 secretion system (T3SS), effector proteins, and the transcriptional regulator SsrB, which positively regulates all the SPI-2 genes. EutR was shown to regulate these genes by directly binding *ssrB*’s promoter and activating expression (68, 69). In addition to these studies done with a mouse model of infection, the *eut* genes were additionally found to promote infection in a *Caenorhabditis elegans* model of intestinal infection. Specifically, a *eutR* mutant proliferated to a significantly lower density in the worm intestine. Whether this was due to EA catabolism or to the regulation of other virulence factors by EutR was not determined (70). Effects on *C. elegans* related to EA utilization are not limited to *S*. Typhimurium; a transposon insertion into a gene encoding one of the BMC structural proteins in *E. faecalis* also resulted in attenuated killing of the animal (71). Finally, the *eut* genes in *S*. Typhimurium have been shown to contribute to proliferation in food, specifically milk and eggs (70) and lettuce and cilantro (72). While not a “host niche” in the traditional sense, proliferation in food products may increase the likelihood of transmission.

Most of the studies involving the role of EA utilization in host interactions involved bacteria that infect the intestinal track, some of which also can infect and reside within host immune cells. However, there is evidence the *eut* genes contribute to infection in other host environments. For example, a meta-analysis of sRNAs related to periodontal disease, an oral infection, discovered a positive association between disease progression and EA catabolism (73). Furthermore, the plant pathogen *Erwinia chrysanthemi* upregulated *eutR* during infection, and loss of this gene attenuated disease: specifically, the pathogen was no longer able to disseminate throughout the plant (74).

Overall, there has been major recent progress establishing that the *eut* genes contribute to the infectivity of several different pathogens. Depending on the context, the contribution occurs by EA catabolism, providing the invader with a source of carbon and/or nitrogen, or by regulation of virulence factors by an EA-sensing regulator. Many important questions remain. While EutR’s ability to directly regulate virulence genes beyond the *eut* genes was shown, there is yet no evidence that EutV or EatR is capable of this additional functionality. Another area that has not been investigated is how EA catabolism might affect the host, particularly the immune system. While any effect at all remains hypothetical, possible scenarios can be envisioned. For example, recall that the final product of EA catabolism is acetate. Acetate in the intestine has anti-inflammatory effects that in part are mediated by its binding to G-protein-coupled receptor 43 (GPR43) expressed on immune cells (75). GPR43 signaling promotes IgA production, which in turn helps modulate the immune response in the GI tract (76). While overall this pathway prevents the host from responding too aggressively to the resident microbiome, could EA-induced acetate production by gut pathogens be a mechanism of undermining the innate immune response during infection?

## CONCLUSIONS

Remarkable strides in understanding the biology of ethanolamine utilization have been achieved in the last several years. These include major regulatory insights, such as the posttranscriptional mechanisms governing gene expression in the EutV system, the identification of the EatR regulator and its role in sensing the breakdown product acetaldehyde rather than EA, and finally the discovery that EutR directly regulates virulence genes in addition to the *eut* genes in certain bacteria. From a variety of pathogens and animal models, there is now clear evidence that the *eut* genes and EA catabolism contribute to pathogen infectivity. The details vary, depending on the system under study, but it is evident that ethanolamine allows adaptation to distinct host environments by precisely coordinating the expression of genes involved in processing EA as a nutrient and, in some systems, virulence genes. The dual role of EA as a nutrient and a signal for virulence gene expression has been best characterized in the EutR-containing systems. It will be interesting to see if EutV and EatR also directly regulate more than just the *eut* genes. Another area that has not been investigated is whether EA utilization and the by-products generated have any direct effects on the host and thereby modulate the host-pathogen interaction in this manner.
